# Pulse pressure variation demonstrates dose-dependent response to volume resuscitation in pediatric craniosynostosis repair

**DOI:** 10.1371/journal.pone.0360509

**Published:** 2026-10-14

**Authors:** Phil Y. Yao, Miles R. Fontenot, Casey A. Quinlan, Vikas N. O’Reilly-Shah

**Affiliations:** 1 Department of Anesthesiology & Pain Medicine, University of Washington, Seattle, Washington, United States of America; 2 Division of Anesthesiology & Pain Medicine, Seattle Children’s Hospital, Seattle, Washington, United States of America; Universiti Sains Malaysia, MALAYSIA

## Abstract

**Background:**

Restoring intravascular volume in the face of hemorrhagic blood loss is a fundamental skill critical to the practice of anesthesiology. Predictors of fluid responsiveness in pediatric patients remain a challenge. Pulse pressure variation (PPV) is a valuable predictor of volume responsiveness in adults, but the utility in pediatric resuscitation remains controversial. Surgical correction of craniosynostosis occurs early in childhood and is commonly associated with significant, rapid blood loss intraoperatively. This study examines the relationship between blood product boluses with PPV and mean arterial pressure (MAP).

**Methods:**

A retrospective, single center analysis of pediatric patients undergoing open cranial vault expansion over a four-year period was performed. Descriptive variables, blood product administrations, MAP, and PPV were extracted and analyzed using a linear mixed-effects model to characterize the association between blood product administration with MAP and PPV. To account for non-independence of repeated measures, the patient was modeled as a random effect with bolus events nested within the patient variable.

**Results:**

A total of 798 bolus events from 111 patients met criteria. Patient ages ranged from 1–23 months (mean 10 months, SD 4.6 months). Transfusion of blood products was associated with increase in MAP by 1.60 mmHg [95% CI: 1.14, 2.06] and reduction in PPV by 0.82 percentage points [95% CI: 0.59, 1.06] (both p < 0.001). Notably, the magnitude of PPV reduction was dose-dependent: for each additional 10 mL/kg administered, post-bolus PPV decreased by an additional 0.4 percentage points [95% CI: 0.02, 0.07] (p = 0.002). In contrast, the MAP increase was not volume-dependent (interaction p = 0.918).

**Conclusion:**

This retrospective analysis of pediatric craniosynostosis repair cases at a single institution suggests that PPV is associated with a dose-dependent response to volume resuscitation, while MAP showed a fixed-magnitude response. These preliminary findings warrant further study in multicenter validity.

## Introduction

Restoring intravascular volume in the face of hemorrhagic blood loss is a fundamental skill critical to the practice of anesthesiology. Optimization of intravascular volume preserves cardiac output and is a foundation principle of hemodynamic resuscitation. The deleterious consequences of inadequate resuscitation and hypovolemia lead to impaired stroke volume, suboptimal cardiac output, and risk for impaired end-organ perfusion. Conversely, care must be taken to avoid excessive fluid administration, which may result in potentially harmful fluid overload [1]. In adult populations, goal-directed fluid therapy is associated with improved post-operative outcomes, including decreased incidence of pulmonary edema, lower risk surgical site infections, and faster gastrointestinal recovery [2–4]. While historic practices of volume resuscitation in pediatric patients relied on weight-based fluid administration, more recent evidence has also demonstrated a benefit of goal-directed fluid therapy in pediatric patients because fluid overload in pediatric patients is associated with increased in-hospital mortality, prolonged mechanical ventilation, and increased incidence of acute kidney injury [5].

Predicting which patients may benefit from volume resuscitation is non-trivial, particularly in pediatric patients, where the physiological response to hypovolemia and fluid administration differs from adults [6]. Many hemodynamic variables have been studied as predictors of fluid responsiveness. In adult populations, pulse pressure variation (PPV), a dynamic variable that reflects the changes in arterial waveform during mechanical ventilation, has emerged as a highly accurate predictor of volume responsiveness across a variety of surgical and critical care settings [7,8]. However, in pediatric populations, the utility of PPV remains controversial in part due to differences in thoracic anatomy and vascular compliance. Some studies suggest that PPV may help guide volume resuscitation, while others have found limited predictive value [9,10].

Renner et al. demonstrated that PPV significantly correlated with stroke volume index changes in infants undergoing congenital heart surgery [11]. Similarly, Han et al. found PPV predictive in infants undergoing ventricular septal defect repair via both sternotomy and thoracotomy [12]. Conversely, other studies have reported limited predictive value for PPV in neurosurgical and thoracoscopic procedures, raising questions about the reliability of PPV across different surgical contexts in pediatric patients [13,14].

In our practice as pediatric anesthesiologists at Seattle Children’s Hospital, we commonly face intraoperative hemorrhage and the need for volume resuscitation when caring for infants and toddlers undergoing open cranial vault expansion. Correction of congenital craniosynostosis typically takes place in infancy or shortly thereafter. This surgical procedure is typically facilitated via bicoronal incision and surgical osteotomies. Intraoperative blood loss is typically anticipated to be significant and at times brisk despite routine use of antifibrinolytics and surgical hemostatic agents [15]. Further complicating volume resuscitation in these cases, blood loss is often near-continuous and difficult to quantify. Within our pediatric anesthesiologist cohort, there is clinical consensus that PPV has considerable value during open craniosynostosis repairs to guide volume resuscitation intraoperatively in an effort to target goal-directed fluid therapy. During these procedures, an increase in PPV is presumed to indicate hypovolemia, prompting volume resuscitation.

The aim of this study was to investigate the relationship between intraoperative blood product volume administration and subsequent change in PPV and mean arterial pressure (MAP) in a relatively homogenous patient population of infants and toddlers undergoing open cranial vault expansion. We hypothesized that following administration of a blood product bolus, PPV would decrease and MAP would increase, suggesting effective volume resuscitation.

## Materials and methods

This observational study followed the Reporting of studies conducted using Observational Routinely collected health Data (RECORD) extension of the Strengthening of Observational Studies in Epidemiology (STROBE) guidelines [16]. Following approval on March 6, 2025 by the Seattle Children’s Institutional Review Board for STUDY00005289, a retrospective analysis of data from infants and toddlers who underwent open cranial vault expansion at Seattle Children’s Hospital between January 1, 2021 and December 31, 2024 was performed. The data was generated during routine anesthetic care and captured in the electronic anesthetic records, then transmitted to the Multicenter Perioperative Outcomes Group (MPOG) for use in clinical research and quality improvement, as previously described [17,18]. The dataset was extracted on March 19, 2025, and is classified as a limited dataset per United States Federal Regulations as it contains dates and times of service, which are apart of the 18 standard HIPAA identifiers.

For each of the extracted cases, the following data were extracted using the MPOG Data Direct tool and analyzed with R: patient demographics (age, sex, height, weight, and American Society of Anesthesia Physical Status classification); time-stamped hemodynamic variables: pulse pressure variation and mean arterial pressure; and, volume and timing of intraoperative blood product boluses. MPOG phenotypes were used to ensure the highest quality of data extraction. Cases were immediately excluded if anesthetic records demonstrated absence of transfusion of packed red blood cells (pRBCs) and/or fresh frozen plasma (FFP) or documented only a single bolus of blood product with volumes greater than 200 ml, as this likely reflects inaccurate temporal documentation.

To assess the physiological response to blood product administration, the data was organized into 5-minute windows before and after each discrete bolus. Discrete blood product boluses (pRBCs and FFP) documented with the same timestamp were summed and treated as a single combined bolus. If multiple boluses were given within the same 10-minute interval, they were aggregated and analyzed as a single combined bolus using the start time of the first bolus and end time of the last bolus. Combined boluses exceeding 200 mL were also excluded.

A total of 188 craniosynostosis repair cases in patients less than 2 years of age were performed over the four-year study period (January 2021-December 2024) at this single institution. Of these, 17 cases were excluded from analysis because no blood product (pRBCs or FFP) was administered; the vast majority (16/17) of these excluded cases represent endoscopic repair of sagittal craniosynostosis, which is associated with substantially less blood loss. One case involving platelet transfusion for pre-existing thrombocytopenia rather than volume resuscitation was also excluded. From an initial 1346 bolus events, single boluses exceeding 200 mL (suggesting inaccurate temporal documentation) were also excluded. Boluses charted within 5 minutes were combined, and combined boluses exceeding 200 mL were also excluded.

A total of 798 bolus events from 111 patients was included in the final analysis. For each bolus event, the pre-bolus PPV and MAP values were calculated as the mean of all values recorded in the 5 minutes preceding the bolus timestamp. Post-bolus values were similarly calculated as the mean of all values recorded in the 5 minutes following the bolus. Changes in PPV (ΔPPV) and MAP (ΔMAP) were calculated as the difference between post-bolus and pre-bolus mean values.

Linear mixed-effects modeling was performed using *lme4* and *lmerTest* packages in R. To account for the repeated-measures hierarchy of paired (Pre vs Post) measurements within each bolus epoch and the multiple bolus events for each individual patient, we specified random intercepts for both patient and bolus event nested within patients. Paired observations were explicitly linked at the lowest level of the model to account for baseline variability across distinct bolus events and patients. Fixed effects included time category (Pre or Post), weight-based bolus volume, and their two-way interaction. Model fit and linear regression assumptions were confirmed via inspection of residual-vs-fitted and normal Q-Q plots. All analyses were conducted using RStudio version 2025.09.0 Build 387 running R version 4.4.2 standard statistical software.

## Results

This study reports on 798 blood product bolus events from 111 patients included in the final analysis. The patients’ ages ranged from 1 to 23 months, with a mean age of 10 months (SD 4.6 months). A total of 75 (67.6%) patients were male, and the mean patient weight was 9.1 kg (SD 2.1 kg) and height was 71.9 cm (SD 7.4 cm). Most patients (60%) were classified as ASA physical status 2. The mean transfused volume of pRBCs was 35.9 (SD 21.7) mL/kg and mean transfused volume of FFP was 31.5 (SD 19.0) mL/kg (Table 1).

**Table 1:** Patient demographics.

Characteristics (mean (SD))	Overall	Excluded	Included
	N = 171	N = 60	N = 111
**Age (month)**	10.2 (4.8)	10.6 (5.0)	10.0 (4.6)
**Male Sex (%)**	117 (68.4%)	42 (70.0%)	75 (67.6%)
**Height (cm)**	72.6 (7.3)	74.1 (7.0)	71.9 (7.4)
**Weight (kg)**	9.2 (2.0)	9.4 (2.0)	9.1 (2.1)
**ASA PS Classification (%)**			
1	32 (18.7%)	10 (16.7%)	22 (19.8%)
2	100 (58.5%)	33 (55.0%)	67 (60.4%)
3	39 (22.8%)	17 (28.3%)	22 (19.8%)
**Packed Red Blood Cell Volume (mL/kg)**	34.9 (19.2)	33.0 (13.1)	35.9 (21.7)
**Fresh Frozen Plasma Volume (mL/kg)**	30.9 (16.6)	29.6 (10.8)	31.5 (19.0)

A linear mixed-effects model was used to analyze the PPV and MAP values that were extracted from the 5 minutes before and 5 minutes after bolus administration. These values were used to calculate mean PPV and MAP for pre- and post- bolus, which along with bolus volume in mL/kg were modeled as fixed effects. Patient ID was modeled as a multilevel random effect with bolus events nested within patient ID. The results of linear mixed-effects model analysis revealed that blood product boluses significantly increased MAP by 1.60 mmHg [95% CI: 1.14, 2.06] and reduced PPV by 0.82% [95% CI: 0.59, 1.06] (both p < 0.001). Notably, the magnitude of PPV reduction was dose-dependent: for each additional 1 mL kg-1 administered, post-bolus PPV decreased by an additional 0.04 percentage points [95% CI: 0.02, 0.07] (p = 0.002) (Fig 1). In contrast, the MAP increase was not volume-dependent (interaction p = 0.918); boluses produced a consistent ~1.6 mmHg increase in MAP irrespective of volume administered.

**Fig 1:**
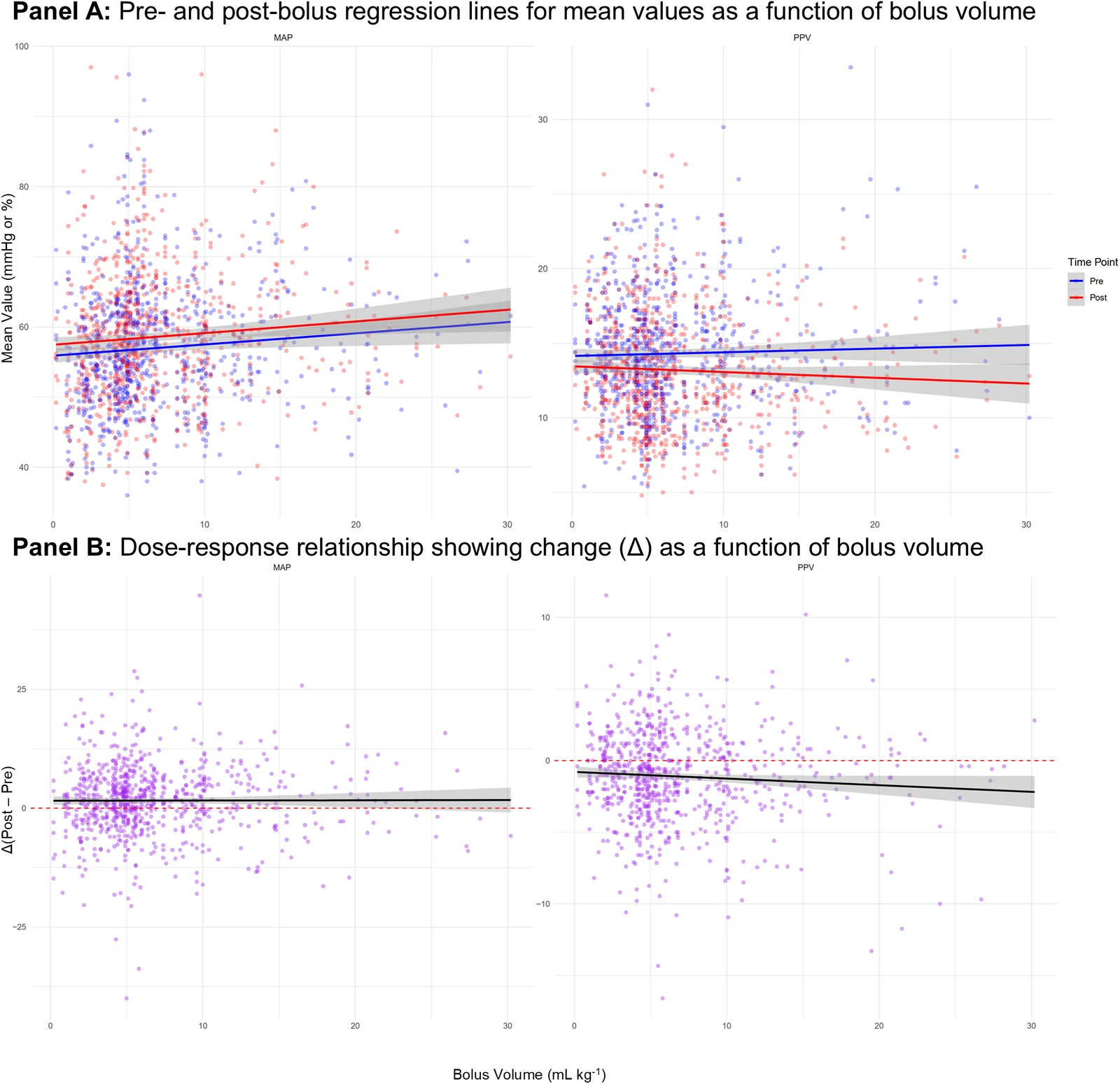
Effect of blood product bolus volume on Mean Arterial Pressure (MAP, mmHg) and Pulse Pressure Variation (PPV, %) in craniosynostosis repair cases in pediatric patients. Blood product boluses increased MAP by 1.60 mmHg [95% CI: 1.14, 2.06] and reduced PPV by 0.82% [95% CI: 0.59, 1.06] (both p < 0.001). The increase in MAP was not volume-dependent (p = 0.918), whereas the decrease in PPV was volume-dependent: for each additional 1 mL kg-1 administered, post-bolus PPV decreased by an additional 0.04% [95% CI: 0.02, 0.07] (p = 0.002).

## Discussion

The results of this study demonstrate the hypothesized relationship between blood product volume transfused and change in PPV and MAP during pediatric craniosynostosis repair. Further, the data show that larger volume of transfused blood products was associated with a greater decrease in PPV, suggesting a corresponding dose-dependent relationship. These findings support further study of PPV as a dynamic, responsive indicator of intravascular volume status and volume responsiveness in pediatric patients.

Importantly, these results provide real-world support for a practice that has become standard at our institution: using PPV to guide administration of blood products during open cranial vault expansion. There are many elements of the anesthetic management of open cranial vault expansion that likely make this patient population ideal for examining the utility of PPV in pediatric patients. Patients undergoing open cranial vault expansion are uniformly mechanically ventilated, heavily monitored, and frequently experience ongoing and significant blood loss, creating ideal conditions for changes in PPV to be clinically meaningful.

Importantly, previous validation studies for the utility of PPV note the importance of controlled ventilation, generous tidal volumes (typically > 7 cc/kg), and a standardized PEEP of 5 cmH2O [8,11]. In our current study, we did not account for mode of ventilation, variations in tidal volume or positive end-expiratory pressure (PEEP). However, because management of patients undergoing open cranial vault expansion typically demands relative hyperventilation to aid in cerebral relaxation and mitigate hemorrhage, it is most common that patients receive controlled ventilation with generous tidal volumes to achieve a targeted end-tidal carbon dioxide near 30 mmHg. Elevated PEEP is also generally uncommon in this subset of patients. Common practice at our institution is to apply a PEEP of 5 cmH2O routinely. Coincidentally, these conditions approximate validation conditions for utility of PPV in adults.

Notably, patient positioning for open cranial vault repair may include supine for frontoorbital advancements or prone positioning for posterior vault expansion. In the current study, patient positioning was not analyzed. Future studies in this patient population should consider exploration of the impact of this variable in pediatric patients.

We note that while the result of this study is statistically significant, the relatively modest increase in MAP by 1.60 mmHg [95% CI: 1.14, 2.06] and reduction in PPV by 0.82 percentage points [95% CI: 0.59, 1.06] (both p < 0.001) may raise the question of clinical significance. However, we hypothesize that the magnitude of effect is likely underestimated in the current study as a function of the retrospective nature of the study design. The current analysis employed multilevel mixed-effects modeling to account for clustering within patients (who may have received multiple boluses), but does not capture provider-level variability in practice patterns, including timing of charting volume administered, mechanism for administering boluses (by syringe versus to gravity), or threshold PPV at which volume is administered. Additionally, combining multiple boluses within a short time frame could introduce noise into the analysis, potentially masking more discrete relationships. It is a limitation of retrospective analysis that could be addressed by a prospective study with tight controls on data recording.

The modest magnitude of change in MAP and PPV in response to volume administration may also reflect the influence of other variables on the hemodynamic response to volume administration. These factors may include baseline cardiovascular function (though most typically normal in this patient population), anesthetic depth, ongoing surgical blood loss, or administration of vasoactive medications. Notably, co-administration of calcium, which is known to affect hemodynamic variables considerably, is of critical importance during rapid transfusion. Finally, patients undergoing open cranial vault reconstruction are also recognized to be at exquisitely elevated risk of venous air embolism (VAE). While we routinely monitor for VAE with the use of a precordial doppler, entrainment of clinically significant volumes of air may also have a substantial effect on hemodynamic measures. Fortunately, this occurrence is infrequent.

While the sample size in this analysis is relatively robust, data from a single center may limit generalizability. Future work should focus on prospective validation of these findings, including more granular analysis of bolus timing and composition (e.g., red blood cells vs. plasma), the role of vasoactive agents, ventilatory parameters, patient positioning, and PPV thresholds for guiding resuscitation. An analysis of PPV values in epochs without blood product administration as control periods would strengthen causal inference. A multicenter study using the MPOG network would improve generalizability and statistical power.

## Conclusion

This study supports the use of pulse pressure variation (PPV) as a dynamic and responsive tool for guiding volume resuscitation during pediatric craniosynostosis repairs. Blood product boluses were associated with dose-dependent decreases in PPV and dose-independent increases in MAP. These findings suggest that PPV can aid intraoperative decision-making regarding volume responsiveness in this unique surgical population.

Given the limitations of retrospective, single-center data, future research should focus on multicenter collaboration and prospective study design. Establishing evidence-based thresholds for PPV-guided resuscitation could improve hemodynamic management and outcomes in high-risk pediatric surgeries. Until then, PPV appears to be a useful adjunct for intraoperative volume assessment in craniosynostosis procedures, when used in the appropriate clinical context.
